# Genomic diversity and admixture patterns among six Chinese indigenous cattle breeds in Yunnan

**DOI:** 10.5713/ajas.18.0605

**Published:** 2019-01-02

**Authors:** Rong Li, Chunqing Li, Hongyu Chen, Xuehong Liu, Heng Xiao, Shanyuan Chen

**Affiliations:** 1 School of Life Sciences, Yunnan University, Kunming, Yunnan 650500, China; 2 National Demonstration Center for Experimental Life Sciences Education, Yunnan University, Kunming Yunnan 650500, China; 3 Faculty of Animal Science and Technology, Yunnan Agricultural University, Kunming, Yunnan 650221, China

**Keywords:** *Bos taurus*, *Bos indicus*, Heterozygosity, Population Structure, Single Nucleotide Polymorphisms

## Abstract

**Objective:**

Yunnan is not only a frontier zone that connects China with South and Southeast Asia, but also represents an admixture zone between taurine (*Bos taurus*) and zebu (*Bos indicus*) cattle. The purpose of this study is to understand the level of genomic diversity and the extent of admixture in each Yunnan native cattle breed.

**Methods:**

All 120 individuals were genotyped using Illumina BovineHD BeadChip (777,962 single nucleotide polymorphisms [SNPs]). Quality control and genomic diversity indexes were calculated using PLINK software. The principal component analysis (PCA) was assessed using SMARTPCA program implemented in EIGENSOFT software. The ADMIXTURE software was used to reveal admixture patterns among breeds.

**Results:**

A total of 604,630 SNPs was obtained after quality control procedures. Among six breeds, the highest level of mean heterozygosity was found in Zhaotong cattle from Northeastern Yunnan, whereas the lowest level of heterozygosity was detected in Dehong humped cattle from Western Yunnan. The PCA based on a pruned dataset of 233,788 SNPs clearly separated Dehong humped cattle (supposed to be a pure zebu breed) from other five breeds. The admixture analysis further revealed two clusters (*K* = 2 with the lowest cross validation error), corresponding to taurine and zebu cattle lineages. All six breeds except for Dehong humped cattle showed different degrees of admixture between taurine and zebu cattle. As expected, Dehong humped cattle showed no signature of taurine cattle influence.

**Conclusion:**

Overall, considerable genomic diversity was found in six Yunnan native cattle breeds except for Dehong humped cattle from Western Yunnan. Dehong humped cattle is a pure zebu breed, while other five breeds had admixed origins with different extents of admixture between taurine and zebu cattle. Such admixture by crossbreeding between zebu and taurine cattle facilitated the spread of zebu cattle from tropical and subtropical regions to other highland regions in Yunnan.

## INTRODUCTION

Cattle are one of the most important livestock species in many aspects. Archaeological and genetic evidence suggested that modern domestic cattle descended from different aurochs populations [1–3], resulting in two main types - humpless/taurine cattle (*Bos taurus*) and humped/zebu cattle (*Bos indicus*) [4]. Since domestication, taurine and zebu cattle each migrated out from its own domestication center, subsequently met with each other and hybridized at some contact regions such as Arabian Peninsula [5] and Southeast Asia [6]. Yunnan is not only a frontier zone that connects China with South and Southeast Asia, but also represents an admixture zone between taurine and zebu cattle. Obviously, some individuals of Yunnan local cattle breeds seem to be offspring of crosses between taurine and zebu cattle.

According to China National Commission of Animal Genetic Resources, there are six indigenous cattle breeds (Diqing, Zhaotong, Dengchuan, Dianzhong, Wenshan, and Dehong humped cattle) in Yunnan, Southwestern China. These breeds provide not only useful products such as cowhide, meat and milk to local farmers and communities, but also are used as the main means of transportation in remote villages. It should be emphasized that Dengchuan cattle is the only one local dairy cattle breed in China with high rate of milk production. In addition, these indigenous breeds exhibit excellent characteristics such as tolerance to rough feeding, good adaptability under adverse climatic conditions, and resistance to some animal diseases and parasites [7,8]. In recent years, the implementation of artificial insemination, together with global climate change and frequent outbreaks of emerging animal diseases, has increased the risk of loss of Yunnan native cattle breeds. The number of some pure native breeds has been decreased from ten thousand head in the 20th century to several hundred head in present day [9]. For instance, Dengchuan cattle are already on the brink of extinction, due to crossbreeding with exotic commercial breeds such as Holstein, Simmental and Shorthorn cattle [9]. As a result, it is urgent to understand their genomic background before loss or extinction. Previous studies on Yunnan native cattle breeds focused mainly on mitochondrial diversity, origins, introgression and phylogeographic structure [7,10,11]. However, information on genomic diversity and admixture patterns among six Chinese indigenous cattle breeds in Yunnan is still very limited.

Recently, high-density single nucleotide polymorphism (SNP) genotyping arrays have become available for bovine genomic analysis, such as Illumina BovineHD BeadChip, which features 777,962 SNPs [12]. The SNP chips have been used to investigate genomic diversity, population structure and admixture patterns of various native cattle breeds from different countries or geographic regions, such as Spain [13], Sudan [14], and Ireland [15]. Herein, we first report a genome-wide survey on six Chinese indigenous cattle breeds in Yunnan using BovineHD Genotyping BeadChip. The aims of this study were to evaluate the level of genomic diversity and quantify the extent of admixture in each breed.

## MATERIALS AND METHODS

### Ethics statement

Ear skin tissues were randomly collected by veterinary practitioners according to relevant international as well as national guidelines and under permission from the Guide for the Care and Use of Laboratory Animals of Yunnan University. All experimental procedures used in this study were approved by the Institutional Animal Care and Use Committee (IACUC) of the Yunnan University (ynucae 20140011).

### Samples collection and genomic DNA extraction

A total of 120 individuals were collected from six officially recognized Chinese indigenous cattle breeds in Yunnan, including Diqing cattle (n = 20), Zhaotong cattle (n = 20), Dengchuan cattle (n = 20), Dianzhong cattle (n = 20), Wenshan cattle (n = 20), and Dehong humped cattle (n = 20) (Table 1). The 120 samples contained 50 males and 70 females, sampled from remote villages, where artificial insemination was not practiced. Care was taken to avoid sampling related individuals, based on information provided by local farmers. A tiny piece of ear skin tissue for each individual was cut by trained veterinarians with the permission and in presence of the owners and deposited in tube with pure ethanol. Genomic DNA was extracted from ear skin tissues using the DNeasy Blood & Tissue kit (QiaGen, Hilden, Germany). The DNA concentration and quality were checked with NanoDrop 2000 spectrophotometer (Thermo Scientific, Wilmington, DE, USA).

### Genotyping and quality control

All 120 individuals were genotyped using Illumina BovineHD BeadChip (777,962 SNPs). The overall genotyping rate was 99.3%. The SNPs were mapped to the UMD 3.1 bovine genome assembly [16]. After excluding unmapped SNPs (1,735) and those on X (39,367), Y (1,224), and mitochondrial (343) chromosomes, a total of 735,293 autosomal SNPs was used for quality control procedures. The SNPs with a call rate of less than 90%, minor allele frequency (MAF) lower than 0.05 and SNPs whose genotypes were not in Hardy-Weinberg equilibrium (p<0.001) were excluded for further analyses [17]. Moreover, to avoid biases caused by linkage disequilibrium (LD), we also pruned SNPs for LD using the command ‘indep (50 5 0.5)’ [18] in PLINK v1.07 [19].

### Data analysis

To estimate the level of genomic diversity in each breed, observed heterozygosity (*Ho*), expected heterozygosity (*He*), and inbreeding coefficient (*f*) were calculated using PLINK v1.07 [19]. Heterozygosity estimates are sensitive to various ascertainment biases when SNPs discovered in one breed are used to genotype other breeds [18,20]. Analyses of diversity indexes and genetic relationships based on ascertained SNP data would produce false positive results [21]. Previous studies demonstrated that pruning of SNPs in high LD would reduce the influence of ascertainment biases [22,23]. Therefore, we used SNP datasets after quality control and LD-pruning (by removing closely associated SNPs) to evaluate the influence of ascertainment biases on diversity indexes.

Furthermore, to discern genetic relationships among breeds, principal component analysis (PCA) was assessed using SMARTPCA program implemented in EIGENSOFT v6.1.4 [24]. The software ADMIXTURE v1.30 [25] was used to reveal admixture patterns among breeds. The corresponding cross-validation error value of cluster (*K* = 1 to 6) was calculated in ADMIXTURE v1.30. The optimal value of *K* would exhibit a low cross-validation error compared with those of other *K* values. The graphical representation of PCA and admixture patterns were depicted using ggplot2 function in R software package [26].

## RESULTS

### Level of polymorphisms within breeds

After quality control, 130,663 SNPs were excluded, in which 11,331 showed SNP call rate of less than 0.90, 109,315 had MAF less than 0.05 and 10,017 significantly deviated from Hardy-Weinberg equilibrium (p<0.001), we finally obtained 604,630 SNPs for subsequent analyses (Table 1). The excluded highest number of SNPs (251,637) that showed a MAF of less than 0.05 was found in Dehong humped cattle, while the excluded lowest number of SNPs (82,819) was observed in Zhaotong cattle. Additionally, all samples passed the pruned SNPs for LD, and obtained a dataset of 233,788 SNPs for genomic diversity, PCA and ADMIXTURE analyses.

**Figure 1:**
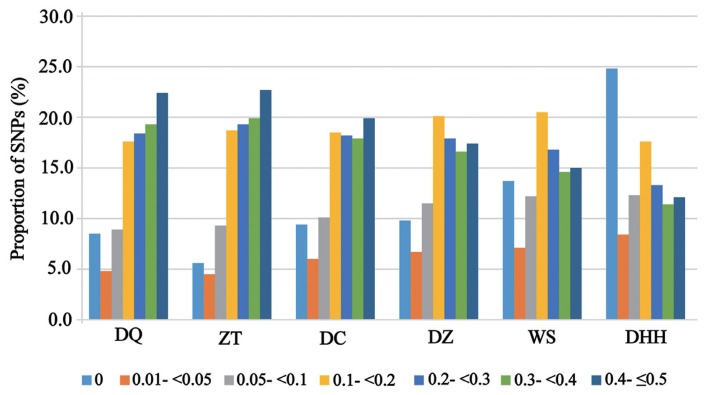
The minor allele frequency (MAF) distribution in each indigenous cattle breed. Breed abbreviations as follows: DQ, Diqing cattle; ZT, Zhaotong cattle; DC, Dengchuan cattle; DZ, Dianzhong cattle; WS, Wenshan cattle; DHH, Dehong humped cattle.

When MAF greater than or equal to 0.4, Zhaotong (22.7%) and Dehong humped (12.1%) cattle showed the highest and lowest proportion of SNPs, respectively. In contrast, when MAF = 0, Zhaotong cattle (5.6%) showed the lowest proportion of SNPs, while Dehong humped cattle showed the highest proportion of SNPs (24.8%) (Figure 1). The 88.3% (on average) of the SNP markers in Diqing and Zhaotong cattle were polymorphic (MAF≥0.05). The percentage of polymorphic SNP (%P) detected in each breed varied from 66.9% in Dehong humped cattle to 89.9% in Zhaotong cattle (Table 2).

### Breed genomic diversity and ascertainment bias

**Figure 2:**
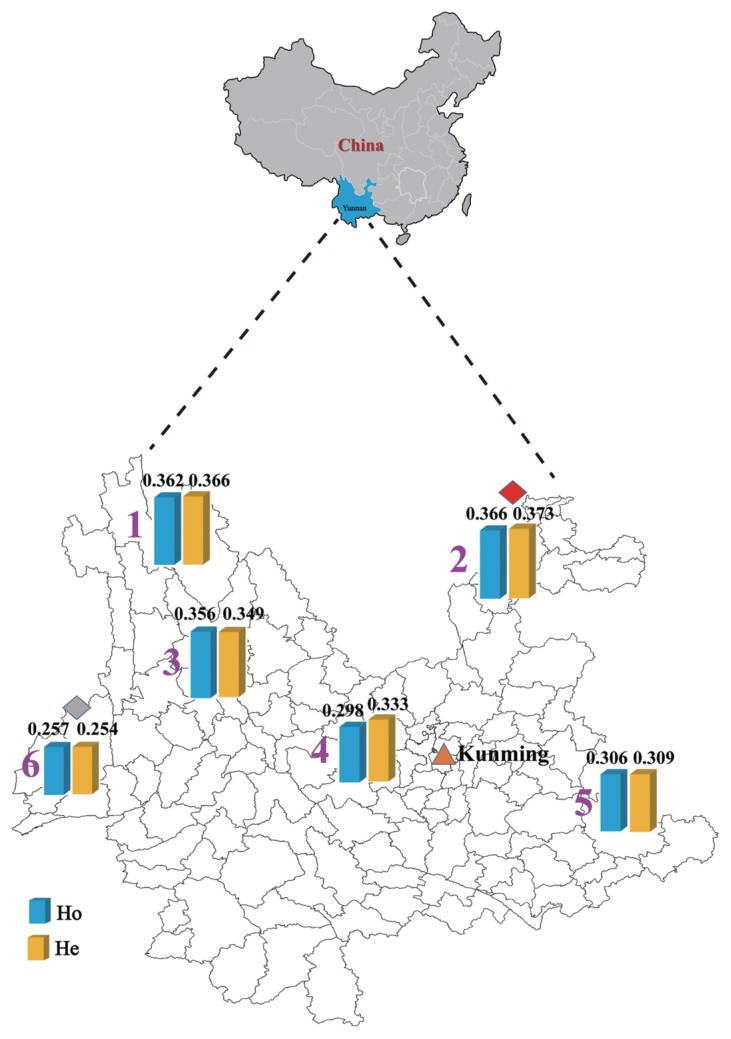
Geographic distribution of genomic diversity of six Yunnan native cattle breeds based on 604,630 single nucleotide polymorphisms. Blue represents observed heterozygosity (*Ho*) and yellow represents expected heterozygosity (*He*). Number notes: 1, Diqing cattle; 2, Zhaotong cattle; 3, Dengchuan cattle; 4, Dianzhong cattle; 5, Wenshan cattle; 6, Dehong humped cattle.

Here we used two different datasets to assess the effect of ascertainment biases on diversity indexes of each breed. Using a dataset of 604,630 SNPs, the average *Ho* ranged from 0.257 in Dehong humped cattle (Western Yunnan) to 0.366 in Zhaotong cattle (Northeastern Yunnan). Similarly, using a dataset of 233,788 SNPs, the lowest *Ho* (0.295) was found in Dehong humped cattle, while the highest *Ho* (0.360) was detected in Zhaotong cattle. Interestingly, there existed a declining trend of genomic diversity for these native breeds from Northeastern Yunnan to Western Yunnan (Table 2, Figure 2). In addition, when using LD-based pruning dataset (233,788 SNPs), we found that heterozygosity values were slightly reduced in Diqing, Zhaotong, and Dengchuan cattle, but were slightly increased in Dianzhong, Wenshan, and Dehong humped cattle. The inbreeding coefficients were also estimated based on two datasets as well. The results indicated that the minimum level of inbreeding was in Dengchuan cattle (−0.022 for 604,630 SNPs, −0.024 for 233,788 SNPs) and the maximum level of inbreeding was in Dianzhong cattle (0.055 for 604,630 SNPs, 0.053 for 233,788 SNPs) (Table 2).

### Principal component and Admixture analysis

**Figure 3:**
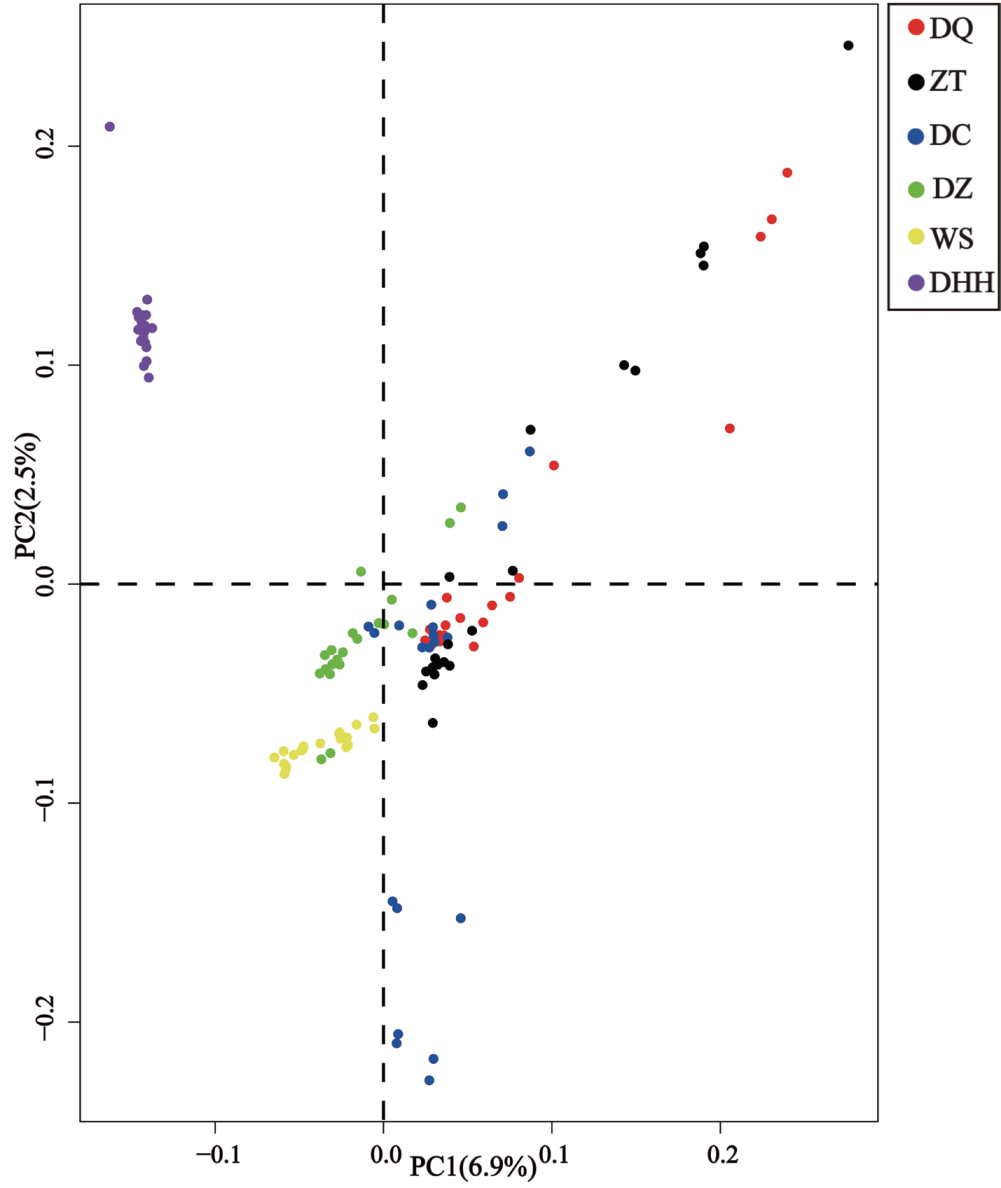
Principal component analysis (PCA) of six Yunnan native cattle breeds based on a pruned dataset of 233,788 single nucleotide polymorphisms. The first (PC1) and second (PC2) components were plotted. Breed abbreviations as follows: DQ, Diqing cattle; ZT, Zhaotong cattle; DC, Dengchuan cattle; DZ, Dianzhong cattle; WS, Wenshan cattle; DHH, Dehong humped cattle.

The genetic relationships among six Yunnan native cattle breeds revealed by PCA are shown in Figure 3. The first (PC1) and second (PC2) principal components accounted for 6.9% and 2.5% of the total variation, respectively. The PC1 clearly separated Dehong humped cattle from other five breeds. The PC2 did not group the other five breeds into clear-cut clusters, implying that genetic admixture probably existed between other five breeds from different geographic regions in Yunnan.

**Figure 4:**
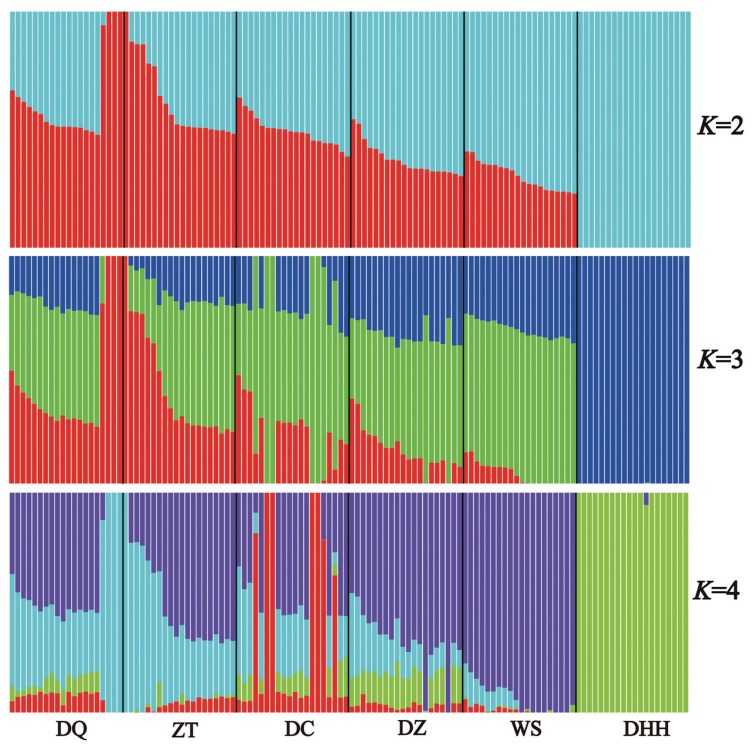
Admixture analysis for six Yunnan native cattle breeds ranging from *K* = 2 to *K* = 4. Breed abbreviations as follows: DQ, Diqing cattle; ZT, Zhaotong cattle; DC, Dengchuan cattle; DZ, Dianzhong cattle; WS, Wenshan cattle; DHH, Dehong humped cattle.

To further examine the extent of admixture in each breed, we executed admixture analysis among breeds and obtained patterns of admixture for each breed (Figure 4). The lowest cross-validation error (0.594) was detected at *K* = 2 (Supplementary Figure S1). When *K* = 2, all six breeds were clustered into taurine and zebu lineages. When looking into details, we found that Dehong humped cattle showed 100% zebu ancestry, while other five breeds showed 49% taurine ancestry and 51% zebu ancestry on average. The overall admixture patterns were in good agreement with those revealed by PCA. Notably, we identified three individuals in Diqing cattle and one individual in Zhaotong cattle appeared to be 100% taurine ancestry. When *K* = 3, all 120 individuals were grouped into three clusters, each for pure taurine, admixed and pure zebu cattle, with 100% zebu ancestry for Dehong humped cattle remained. In addition, when *K* = 4, it showed that Dehong humped cattle (i.e., zebu) component appeared in other five Yunnan native cattle breeds with different percentages. As expected, Dehong humped cattle remained to be pure zebu ancestry (around 100%), being consistent with those found in *K* = 2 and *K* = 3 (Figure 4).

## DISCUSSION

In this study, six Chinese indigenous cattle breeds from Yunnan show a high level of genomic diversity. The heterozygosities (*Ho* = 0.327, *He* = 0.352 for 604,630 SNPs) in Yunnan six native cattle breeds (Table 2) was slightly higher than those reported in Spanish beef cattle breeds (*He* = 0.30) [13] and lower than those in Irish cattle (*Ho* = 0.379) by using bovine high-density SNP chips [15]. Our results revealed that the *Ho* ranged from the lowest in Dehong humped cattle (Western Yunnan) to the highest in Zhaotong cattle (Northeastern Yunnan), showing a declining trend of diversity indexes for six native cattle breeds from Northeastern Yunnan to Western Yunnan (Figure 2), being consistent with that trend revealed by mitochondrial D-loop sequences [11]. Interesting, we found the *Ho* values of Dengchuan (0.356) and Dehong humped cattle (0.257) were higher than those from medium-density bovine SNP chip assay (0.243 in Dengchuan cattle and 0.161 in Dehong humped cattle) [27]. This difference may be mainly attributed to different numbers of SNPs genotyped.

Comparing to other five cattle breeds (Table 2), Dehong humped cattle showed the lower level of polymorphism, MAF and heterozygosities. This might be directly related to ascertainment biases, caused by relatively less SNPs from zebu-type breeds designed in BovineHD SNP chip. This observation and interpretation were in agreement with previous reports [18,28], despite that LD-pruning could minimize the effect of ascertainment biases [22,23]. As expected, pruned SNP dataset slightly increased the level of diversity in Dehong humped cattle than previously unpruned dataset.

The results of the PCA and admixture analyses indicate that other five Yunnan native breeds except for Dehong humped cattle are weakly differentiated. This observation was consistent with previous studies on phylogeographic structure of Yunnan native cattle breeds [11]. The PCA and admixture analyses could separate the breeds into clusters. In this study, the PC1 and admixture analyses clearly separated Dehong humped cattle from other five breeds. This is explained by the fact that Dehong humped cattle is a pure zebu breed (100% zebu ancestry) as revealed by Li et al [11] and that this breed distributes in Dehong Prefecture (a region close to Myanmar) of Yunnan Province, belonging to tropical and subtropical climatic conditions. These facts indicated that Dehong humped cattle are descendants of zebu-type ancestral cattle without influence of taurine cattle, because taurine cattle poorly adapt in such tropical and subtropical climatic conditions. In addition, when *K* = 2, the admixture analysis separates all six breeds into taurine and zebu lineages, corresponding to two independent ancestries of modern domestic cattle [1,29,30]. When *K* = 3, all samples were grouped into three clusters, each for pure taurine, admixed and pure zebu cattle, which provided clear evidence for hybridization between taurine and zebu cattle [31].

## CONCLUSION

In this study, we found considerable genomic diversity in six Yunnan native cattle breeds except for Dehong humped cattle from Western Yunnan. Our genome-wide analysis further confirmed that Dehong humped cattle is a pure zebu breed with 100% zebu ancestry and no influence from taurine cattle. We also found that five breeds (Diqing, Zhaotong, Dengchuan, Dianzhong, and Wenshan cattle) had admixed origins with different degrees of admixture between taurine and zebu cattle. Such admixture by crossbreeding between zebu and taurine cattle facilitated the spread of zebu cattle from tropical and subtropical regions to other highland regions in Yunnan. Future studies are needed to detect chromosomal segments or genomic regions that have adaptive significance in admixed individuals.

## Supplementary Data



## Figures and Tables

**Table 1:** Breed, sampling locality, number of samples (*n*) and SNPs excluded and remaining after quality control based on 735,293 SNPs

Breed	Geographic distribution	*n*	Excluded SNPs				Remaining SNPs

			SNP CR<0.90	MAF<0.05	HWE	Total	
Diqing (DQ)	Diqing prefecture, Yunnan	20	9,390	97,719	609	105,892	629,401
Zhaotong (ZT)	Ludian county, Yunnan	20	9,538	74,204	589	82,819	652,474
Dengchuan (DC)	Eryuan county, Yunnan	20	10,864	113,470	587	122,864	612,429
Dianzhong (DZ)	Shuangbai county, Yunnan	20	10,264	121,688	906	130,622	604,671
Wenshan (WS)	Guangnan county, Yunnan	20	13,568	153,307	685	164,494	570,799
Dehong humped (DHH)	Mangshi county, Yunnan	20	11,147	243,706	587	251,637	483,656
Total		120	11,331	109,315	10,017	130,663	604,630

SNP, single nucleotide polymorphism; CR, call rate; MAF, minor allele frequency; HWE, Hardy-Weinberg equilibrium (p<0.001).

**Table 2:** The genetic diversity indexes (*Ho* and *He*) and inbreeding coefficient in the studied indigenous cattle populations

Breed	*n*	MAF	%P	604,630 SNPs			233,788 SNPs		
	
				*Ho*	*He*	*f*	*Ho*	*He*	*f*
Diqing (DQ)	20	0.240	86.7	0.362	0.366	0.012	0.357	0.359	0.007
Zhaotong (ZT)	20	0.247	89.9	0.366	0.373	0.020	0.360	0.367	0.018
Dengchuan (DC)	20	0.225	84.6	0.356	0.349	−0.022	0.355	0.347	−0.024
Dianzhong (DZ)	20	0.212	83.5	0.298	0.333	0.055	0.320	0.338	0.053
Wenshan (WS)	20	0.193	77.6	0.306	0.309	0.009	0.318	0.320	0.006
Dehong humped (DHH)	20	0.157	66.9	0.257	0.254	−0.013	0.295	0.291	−0.014
Total	120	0.225	85.1	0.327	0.352	0.071	0.334	0.356	0.061

MAF, minor allele frequency; % P, percentage of polymorphic SNPs in the 735,293 SNP dataset; *Ho*, observed heterozygosity; *He*, expected heterozygosity; *f*, inbreeding coefficient.
