# Supplementary material for: Genomic diversity and admixture patterns among six Chinese indigenous cattle breeds in Yunnan
Source: Asian-Australas J Anim Sci. 2019 Jan 2;32(8):1069–76. doi: 10.5713/ajas.18.0605 (PMC6599958; doi:10.5713/ajas.18.0605)
Supplement: Supplementary file 1 [file ajas-18-0605-suppl.pdf]

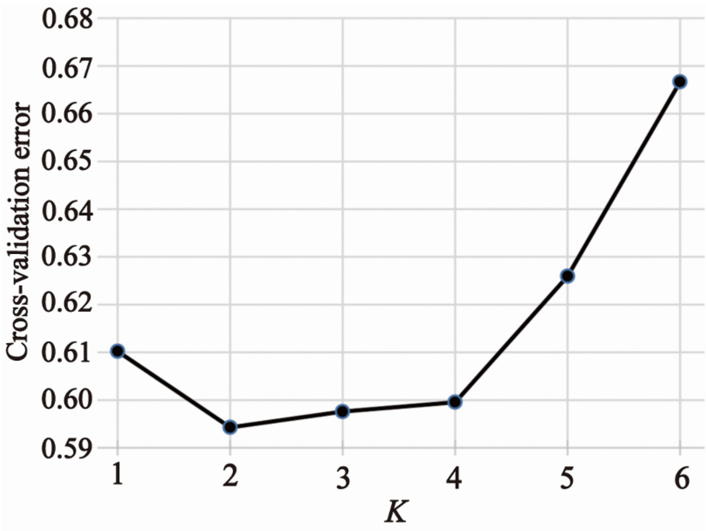

Supplementary Figure S1. Cross-validation error calculated for each value of *K* = 1 to 6 in ADMIXTURE program.
